# Brain perivascular diffusion and cognitive impairment in heart failure with preserved ejection fraction

**DOI:** 10.3389/fmed.2026.1811109

**Published:** 2026-09-15

**Authors:** Tianyi Yu, Chongzhen Duan, Minwen Sun, Yixin Liu, Yuchun Yuan

**Affiliations:** 1 Department of Radiology, Shandong Provincial Hospital Affiliated to Shandong First Medical University, Jinan, China; 2 Shandong Key Laboratory of Multimodal Imaging Artificial Intelligence and Genetics, Jinan, China; 3 Department of Radiology, The Second Affiliated Hospital of Shandong University of Traditional Chinese Medicine, Jinan, China; 4 School of Clinical and Basic Medical Sciences, Shandong First Medical University & Shandong Academy of Medical Sciences, Jinan, China

**Keywords:** cognitive impairment, diffusion tensor image analysis along the perivascular space, glymphatic system, heart failure with preserved ejection fraction, magnetic resonance imaging

## Abstract

**Objective:**

Heart failure with preserved ejection fraction (HFpEF) is increasingly recognized as a systemic cardiovascular disorder that may involve extra-cardiac organs, including the brain. This study aimed to investigate whether brain perivascular diffusivity, assessed using diffusion tensor image analysis along the perivascular space (DTI-ALPS), is associated with cognitive performance, assessed with the Montreal Cognitive Assessment (MoCA) and the Mini-Mental State Examination (MMSE), in patients with HFpEF.

**Materials and methods:**

Thirty-five patients with HFpEF and 20 healthy controls underwent brain diffusion kurtosis imaging and neuropsychological assessment. Left, right, and mean DTI-ALPS indices were calculated for each participant. Group differences were evaluated using independent-samples *t* tests, Mann–Whitney U tests, or chi-square tests as appropriate. Associations between DTI-ALPS indices and cognitive outcomes were examined using Spearman correlation analyses and multivariable linear regression models adjusted for age, sex, and educational level.

**Results:**

Compared with healthy controls, patients with HFpEF had lower MoCA (22.34 ± 3.38 vs. 28.70 ± 1.69; *t* = 9.29, *p* < 0.01) and MMSE scores (median 24.00 vs. 28.50; *Z* = −5.03, *p* < 0.01), together with poorer visuospatial/executive function, attention, language, abstraction, and delayed recall (all *p* < 0.01), whereas naming and orientation did not differ between groups (both *p* > 0.05). The left DTI-ALPS index was lower in patients than in controls (1.64 ± 0.24 vs. 1.77 ± 0.21, *t* = 2.08, *p* = 0.04), whereas the right and mean indices showed no significant group difference (*p* = 0.54 and *p* = 0.16, respectively). After adjustment for age, sex, and educational level, a lower left DTI-ALPS index was independently associated with worse MMSE (*β* = 0.031, *p* = 0.001), MoCA (*β* = 0.021, *p* = 0.007), and delayed recall scores (*β* = 0.050, *p* = 0.009).

**Conclusion:**

HFpEF was associated with cognitive impairment and a left-predominant reduction in perivascular diffusivity as reflected by DTI-ALPS. These findings suggest that DTI-ALPS may be a candidate neuroimaging marker of brain involvement in HFpEF. Given the cross-sectional design, causal and predictive interpretations should be avoided until confirmed by longitudinal multimodal studies.

## 1 Introduction

Heart failure with preserved ejection fraction (HFpEF) is a major chronic cardiovascular syndrome characterized by symptoms and signs of heart failure despite a preserved left ventricular ejection fraction (1). Rather than representing an isolated disorder of diastolic function, HFpEF is now increasingly regarded as a systemic condition driven by endothelial dysfunction, low-grade inflammation, vascular stiffness, and impaired cardiac reserve. Although resting cardiac output may appear relatively preserved, the ability to maintain adequate perfusion during physiological stress may be reduced, potentially affecting vulnerable peripheral organs, including the brain (2).

Growing evidence indicates that cognitive impairment and structural brain alterations are common in patients with chronic heart failure (3). Deficits in executive function, attention, processing speed, and memory have been reported in this population (4), and neuroimaging studies have demonstrated changes such as reduced gray matter volume and white matter abnormalities (5). However, the mechanisms linking HFpEF to brain dysfunction remain incompletely understood, and conventional structural imaging may be insufficiently sensitive to early or subtle brain changes.

The glymphatic system and perivascular pathways play important roles in cerebrospinal fluid (CSF) movement, interstitial solute clearance, and maintenance of brain homeostasis. According to current models, CSF enters the brain along periarterial spaces, exchanges with interstitial fluid, and subsequently drains along perivenous and meningeal/cervical lymphatic routes (6). Impairment of this clearance system may contribute to neurodegeneration by reducing the elimination of neurotoxic solutes and promoting neuroinflammation (7). Although glymphatic dysfunction has been linked to aging and Alzheimer disease (8), its relevance to HFpEF-related cognitive impairment has not been fully explored.

One potential driving factor of glymphatic-perivascular function is cardiovascular pulsatility. Arterial pulsation is thought to facilitate perivascular CSF movement (9). Patients with HFpEF may exhibit persistent hemodynamic abnormalities, arterial stiffness, endothelial dysfunction, and systemic inflammation even in the absence of overt brain atrophy (10, 11). These abnormalities may alter perivascular fluid dynamics and could contribute to cognitive decline (12, 13). However, whether HFpEF is associated with altered perivascular diffusivity, and whether such alterations show hemispheric asymmetry, remains unclear.

The analysis along the perivascular space (ALPS) index, calculated from diffusion MRI, provides a noninvasive surrogate measure of water diffusivity along perivascular spaces near the lateral ventricles. Although the ALPS index does not directly measure glymphatic flow, reduced ALPS values have been reported in several neurological and systemic disorders and may reflect altered perivascular diffusivity. Hemispheric asymmetry in perivascular function may be clinically relevant because the left hemisphere is dominant for language and several complex cognitive functions in most right-handed individuals (14).

In this study, we used diffusion kurtosis imaging-derived DTI-ALPS analysis to assess perivascular diffusivity in patients with HFpEF and healthy controls. Cognition was evaluated with the Montreal Cognitive Assessment (MoCA) and the Mini-Mental State Examination (MMSE). We investigated group differences in left, right, and mean DTI-ALPS indices and examined their associations with global and domain-specific cognitive performance. We hypothesized that HFpEF would be associated with altered perivascular diffusivity and that lower DTI-ALPS values would be related to poorer cognitive performance.

## 2 Methods

Participants were recruited from Shandong Provincial Hospital affiliated to Shandong First Medical University between December 2022 and June 2023. The study included patients with HFpEF and healthy controls. HFpEF was diagnosed according to clinical and echocardiographic criteria, including a left ventricular ejection fraction (LVEF) > 50%. All participants were right-handed. Eligibility criteria included the absence of contraindications to MRI or claustrophobia preventing MRI scanning. Participants were excluded if they had a history of major neurological or psychiatric disorders, including dementia, stroke, brain tumors, or severe psychiatric disease; large cerebrovascular lesions on MRI, such as Fazekas score = 3 or lacunar infarctions; incomplete clinical data; or poor-quality neuroimaging data. Healthy controls had no major cardiovascular or neurological disease.

### 2.1 Clinical data

After overnight fasting, venous blood samples were obtained to measure high-sensitivity C-reactive protein (hs-CRP), fasting plasma glucose (FPG), triglycerides (TG), total cholesterol (TC), and N-terminal pro-brain natriuretic peptide (NT-proBNP). Laboratory tests were performed at Shandong Provincial Hospital affiliated to Shandong First Medical University. All patients underwent standard transthoracic echocardiography in the hospital echocardiography laboratory, and LVEF was recorded as the echocardiographic parameter used in the present analysis.

### 2.2 Neuropsychological examinations

Cognitive performance was assessed using the Montreal Cognitive Assessment (MoCA) and the Mini-Mental State Examination (MMSE). The MoCA includes subdomains assessing visuospatial/executive function, naming, attention, language, abstraction, delayed recall, and orientation (15). The MMSE was used to assess global cognitive function (16). All assessments were performed by trained staff in a quiet environment. The delayed recall subscore of the MoCA, ranging from 0 to 5, was extracted and analyzed as the memory variable.

### 2.3 Magnetic resonance imaging protocol

Brain MRI was performed using a 3.0-T Siemens Prisma scanner equipped with a 64-channel head coil at Shandong Provincial Hospital affiliated to Shandong First Medical University. Participants were positioned comfortably, and foam padding was used to minimize head motion.

Three-dimensional T1-weighted structural images were acquired using a magnetization-prepared rapid acquisition gradient-echo sequence with the following parameters: repetition time = 2,530 ms; echo time = 2.98 ms; inversion time = 1,100 ms; field of view = 256 × 256 mm^2^; flip angle = 7°; and 192 axial slices. Diffusion kurtosis imaging was performed using a spin-echo echo-planar imaging sequence with repetition time = 5,000 ms; echo time = 95 ms; field of view = 224 × 224 mm^2^; matrix = 110 × 110; 74 slices; slice thickness = 2.0 mm; and diffusion weighting in 64 directions with b values of 0, 2,000, and 3,000 s/mm^2^. All images were reviewed by two neuroimaging experts for quality control.

### 2.4 Data preprocessing and DTI-ALPS analysis

Diffusion data were preprocessed using the FMRIB Software Library (FSL). Eddy-current distortions and motion-related artifacts were corrected, nonbrain tissue was removed, and diffusivity and fractional anisotropy maps were generated. Diffusion maps were registered to each participant’s 3D T1-weighted structural image and normalized to Montreal Neurological Institute (MNI) standard space using SPM12. For DTI-ALPS analysis, fixed-size rectangular regions of interest (ROIs) were placed bilaterally at the level of the body of the lateral ventricle on color-coded fractional anisotropy maps. ROIs were positioned in projection-fiber and association-fiber regions corresponding to the corona radiata/superior longitudinal fasciculus, with careful visual inspection to avoid cerebrospinal fluid, gray matter, and visible lesions. The same standardized ROI placement strategy was applied to all participants, and the ROIs were then transferred to the corresponding diffusivity maps to extract directional diffusivities.

The DTI-ALPS index was calculated as [(Dxxproj + Dxxassoc)/2]/[(Dyyproj + Dzzassoc)/2], where Dxxproj and Dxxassoc represent x-axis diffusivity in projection- and association-fiber ROIs, respectively, Dyyproj represents y-axis diffusivity in the projection-fiber ROI, and Dzzassoc represents z-axis diffusivity in the association-fiber ROI. This calculation is based on the anatomical assumption that perivascular spaces around medullary veins are oriented predominantly along the x-axis at this level, whereas the dominant fiber directions of projection and association fibers are orthogonal to that axis. Thus, the numerator reflects water diffusivity along the presumed perivascular direction, while the denominator reduces the influence of background diffusion along the major white-matter fiber directions. A lower DTI-ALPS index was interpreted as reduced perivascular diffusivity. Because DTI-ALPS is an indirect static imaging surrogate rather than a direct measurement of CSF or interstitial fluid flow, its interpretation requires caution.

### 2.5 Statistical analysis

Statistical analyses were performed using SPSS version 22 and R version 4.4.1. Continuous variables were assessed for distributional normality. Normally distributed variables are reported as mean ± standard deviation and were compared using independent-samples *t* tests. Non-normally distributed variables are reported as median and interquartile range and were compared using Mann–Whitney U tests. Categorical variables are summarized as counts and percentages and were compared using chi-square tests or Fisher exact tests when appropriate. Spearman correlation analyses were used to examine associations between DTI-ALPS indices and cognitive scores. Multivariable linear regression models were then used to evaluate the association between the left DTI-ALPS index and cognitive outcomes, with MMSE, MoCA, and memory scores analyzed as separate dependent variables. Models were adjusted for age, sex, and educational level. All statistical tests were two-sided, and *p* < 0.05 was considered statistically significant.

## 3 Results

A total of 50 patients with HFpEF and 30 healthy controls were initially screened. After exclusion for incomplete clinical data, MRI contraindications, severe cerebrovascular lesions, or poor imaging quality, 35 patients with HFpEF and 20 healthy controls were included in the final analysis. The participant selection process is shown in Figure 1.

**Figure 1:**
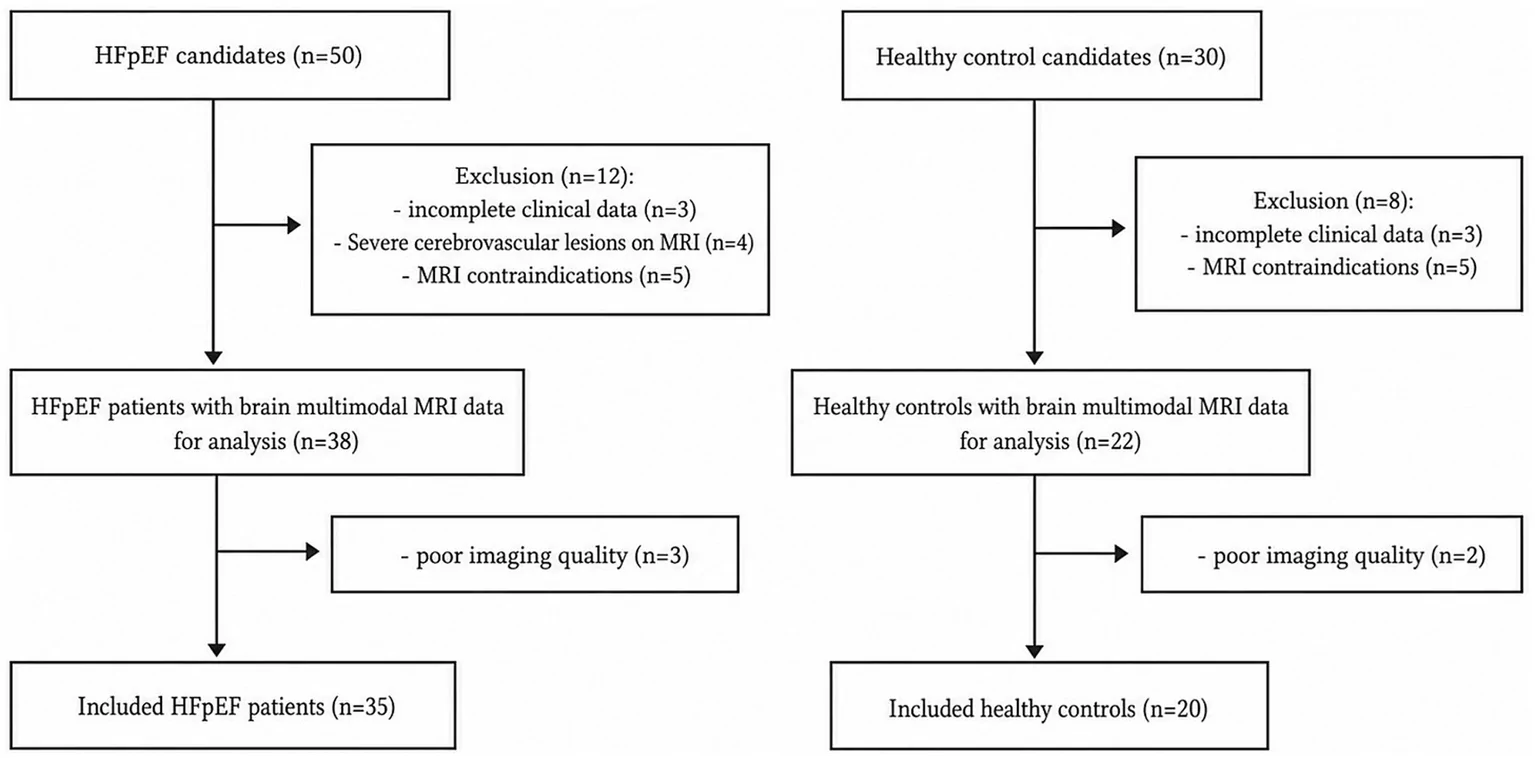
Flowchart of participant recruitment and selection. HFpEF, heart failure with preserved ejection fraction; MRI, magnetic resonance imaging.

### 3.1 Comparison of clinical data between HFpEF group and HCs

Baseline characteristics are summarized in Table 1. The HFpEF and healthy control groups were broadly comparable in age, sex, educational level, body mass index, lipid profile, high-sensitivity C-reactive protein, fasting glucose, smoking history, alcohol consumption, hypertension, hyperlipidemia, and diabetes mellitus (all *p* > 0.05). As expected, NT-proBNP levels were significantly higher in patients with HFpEF than in healthy controls [219.00 (84.65, 428.00) pg/mL vs. 52.45 (21.53, 77.38) pg/mL, *p* < 0.01].

**Table 1:** Demographics and clinical characteristics of patients with HFpEF and healthy controls.

Variables	Total (*n* = 55)	Healthy controls (*n* = 20)	HFpEF patients (*n* = 35)	Statistic	*p*
Age, M (Q₁, Q₃)	63.00 (54.50, 67.50)	58.00 (54.00, 68.00)	64.00 (56.00, 66.50)	*Z* = −0.54	0.59
Male, *n*(%)	33 (60.00)	12 (60.00)	21 (60.00)	*χ*^2^ = 0.00	1.00
Education level, M (Q₁, Q₃)	12.00 (6.00, 12.00)	9.00 (8.25, 12.00)	12.00 (6.00, 15.00)	*Z* = −0.71	0.48
Body mass index, M (Q₁, Q₃)	25.00 (24.10, 26.30)	24.95 (24.08, 26.13)	25.20 (24.20, 27.15)	*Z* = −0.76	0.45
Total cholesterol, M (Q₁, Q₃)	4.38 (3.64, 5.16)	4.39 (3.60, 5.29)	4.37 (3.71, 4.99)	*Z* = −0.72	0.47
Triglycerides, M (Q₁, Q₃)	1.33 (1.05, 1.84)	1.37 (1.04, 2.02)	1.31 (1.06, 1.81)	*Z* = −0.48	0.63
Smoking history, *n*(%)	21 (38.18)	9 (45.00)	12 (34.29)	*χ*^2^ = 0.62	0.43
Alcohol consumption, *n*(%)	23 (41.82)	9 (45.00)	14 (40.00)	*χ*^2^ = 0.13	0.72
Hypertension, *n*(%)	34 (61.82)	13 (65.00)	21 (60.00)	*χ*^2^ = 0.13	0.71
Hyperlipidemia, *n*(%)	18 (32.73)	7 (35.00)	11 (31.43)	*χ*^2^ = 0.07	0.79
Diabetes mellitus, *n*(%)	11 (20.00)	5 (25.00)	6 (17.14)	*χ*^2^ = 0.12	0.73
NT-proBNP (pg/mL), M (Q₁, Q₃)	112.00 (52.45, 371.50)	52.45 (21.53, 77.38)	219.00 (84.65, 428.00)	*Z* = −3.66	<0.01
CRP, M (Q₁, Q₃)	1.25 (0.77, 2.33)	1.60 (0.79, 2.40)	1.23 (0.77, 2.23)	*Z* = −0.24	0.81
Blood glucose, M (Q₁, Q₃)	4.90 (4.49, 5.59)	5.22 (4.68, 5.82)	4.83 (4.35, 5.36)	*Z* = −1.41	0.16

*Z*: Mann–Whitney test, *χ*^2^: Chi-square test, HFpEF: heart failure with preserved ejection fraction, M: median, Q₁: 1st quartile, Q₃: 3rd quartile, NT-proBNP: N-terminal pro-brain natriuretic peptide, CRP: C-reactive protein.

### 3.2 Comparison of cognitive function and DTI-ALPS indices between HFpEF group and HCs

Group comparisons of cognitive function and DTI-ALPS indices are presented in Table 2 and Figure 2. Patients with HFpEF had significantly lower MoCA and MMSE scores than healthy controls (both *p* < 0.01). Domain-level analyses showed poorer visuospatial/executive function, attention, language, abstraction, and memory in the HFpEF group (all p < 0.01), whereas naming and orientation scores did not differ significantly between groups (both *p* > 0.05).

**Table 2:** Comparison of cognitive function and DTI-ALPS indices between healthy controls and patients with HFpEF.

Variables	Total (*n* = 55)	Healthy controls (*n* = 20)	HFpEF patients (*n* = 35)	Statistic	*p*
MoCA score, Mean ± SD	24.65 ± 4.21	28.70 ± 1.69	22.34 ± 3.38	*t* = 9.29	<0.01
MMSE score, M (Q₁, Q₃)	26.00 (23.00, 27.00)	28.50 (27.00, 29.00)	24.00 (20.00, 26.00)	*Z* = −5.03	<0.01
Visuospatial/executive function, M (Q₁, Q₃)	4.00 (2.50, 4.00)	4.50 (4.00, 5.00)	3.00 (2.00, 4.00)	*Z* = −4.49	<0.01
Naming, M (Q₁, Q₃)	3.00 (3.00, 3.00)	3.00 (3.00, 3.00)	3.00 (3.00, 3.00)	*Z* = −0.79	0.43
Attention, M (Q₁, Q₃)	6.00 (4.00, 6.00)	6.00 (6.00, 6.00)	5.00 (3.00, 6.00)	*Z* = −3.87	<0.01
Language, M (Q₁, Q₃)	3.00 (2.00, 3.00)	3.00 (3.00, 3.00)	2.00 (2.00, 3.00)	*Z* = −3.40	<0.01
Abstraction, M (Q₁, Q₃)	2.00 (1.00, 2.00)	2.00 (2.00, 2.25)	2.00 (1.00, 2.00)	*Z* = −2.63	<0.01
Delayed recall (MoCA), M (Q₁, Q₃)	4.00 (2.00, 5.00)	5.00 (5.00, 5.00)	3.00 (1.50, 3.00)	*Z* = −5.47	<0.01
Orientation, M (Q₁, Q₃)	6.00 (5.00, 6.00)	6.00 (5.00, 6.00)	6.00 (5.00, 6.00)	*Z* = −0.76	0.45
DTI-ALPS Left, Mean ± SD	1.68 ± 0.24	1.77 ± 0.21	1.64 ± 0.24	*t* = 2.08	0.04
DTI-ALPS Right, Mean ± SD	1.51 ± 0.22	1.54 ± 0.26	1.50 ± 0.20	*t* = 0.62	0.54
DTI-ALPS Mean, Mean ± SD	1.59 ± 0.21	1.64 ± 0.21	1.56 ± 0.20	*t* = 1.41	0.16

*t*: *t*-test, *Z*: Mann–Whitney *U* test, SD: standard deviation, M: median, Q₁: 1st quartile, Q₃: 3rd quartile, MoCA: Montreal cognitive assessment, MMSE: mini-mental state examination. Memory was defined as the delayed recall subscore of the MoCA.

**Figure 2:**
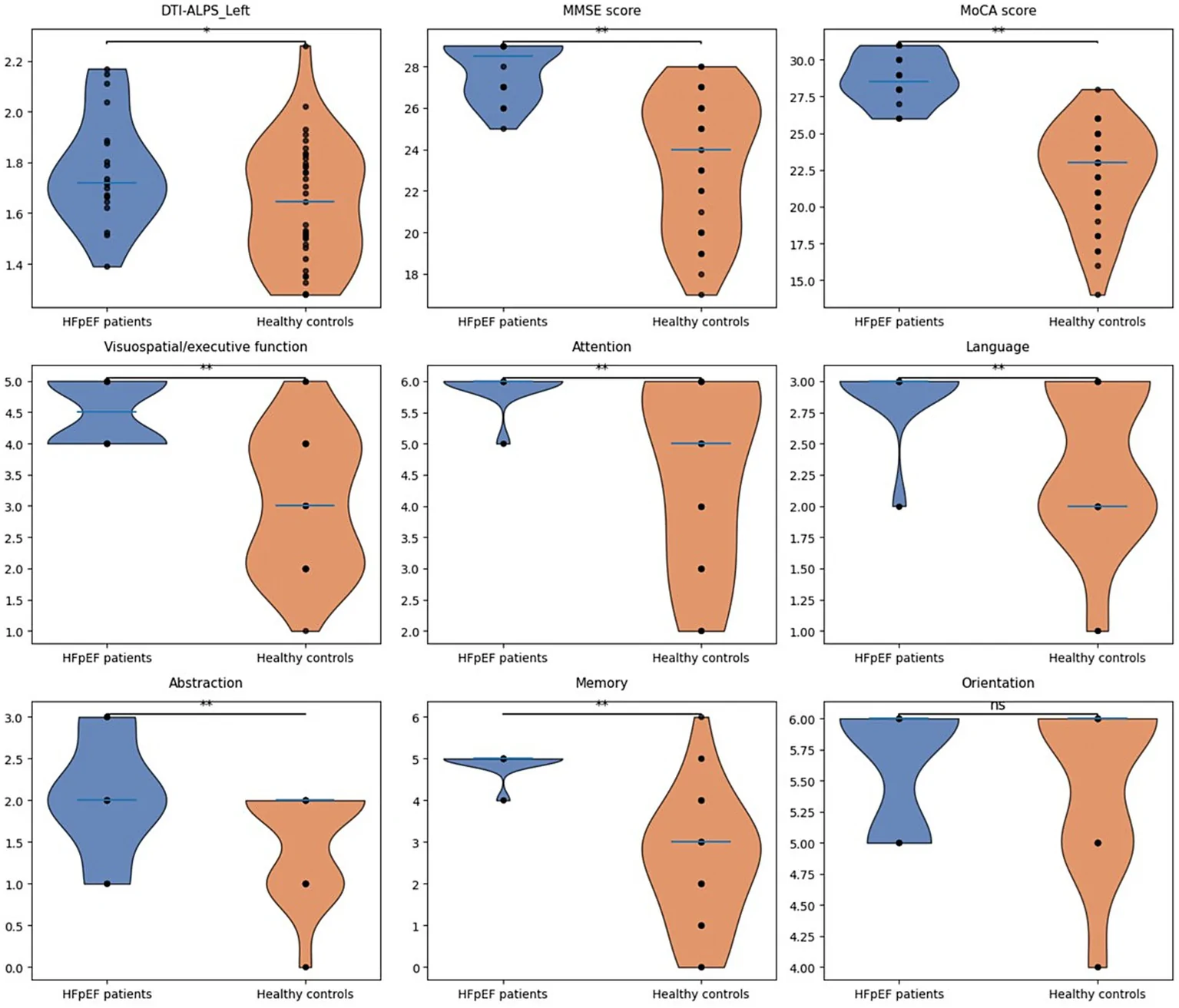
Group differences in DTI-ALPS index and cognitive domain scores between HFpEF patients and healthy controls. DTI-ALPS, diffusion tensor image analysis along the perivascular space; MoCA: Montreal cognitive assessment; MMSE: Mini-Mental State Examination.

For perivascular diffusivity, the HFpEF group showed a significantly lower left DTI-ALPS index than healthy controls (*p* = 0.04). In contrast, group differences in the right DTI-ALPS and mean DTI-ALPS indices were not statistically significant (both *p* > 0.05).

### 3.3 Correlation analysis

Spearman correlation analyses were performed to explore the relationships between DTI-ALPS indices and cognitive performance in patients with HFpEF (Figure 3). The left DTI-ALPS index was positively correlated with MMSE (*r* = 0.32, *p* < 0.05), MoCA (*r* = 0.28, *p* < 0.05), and memory scores (*r* = 0.33, *p* < 0.05).

**Figure 3:**
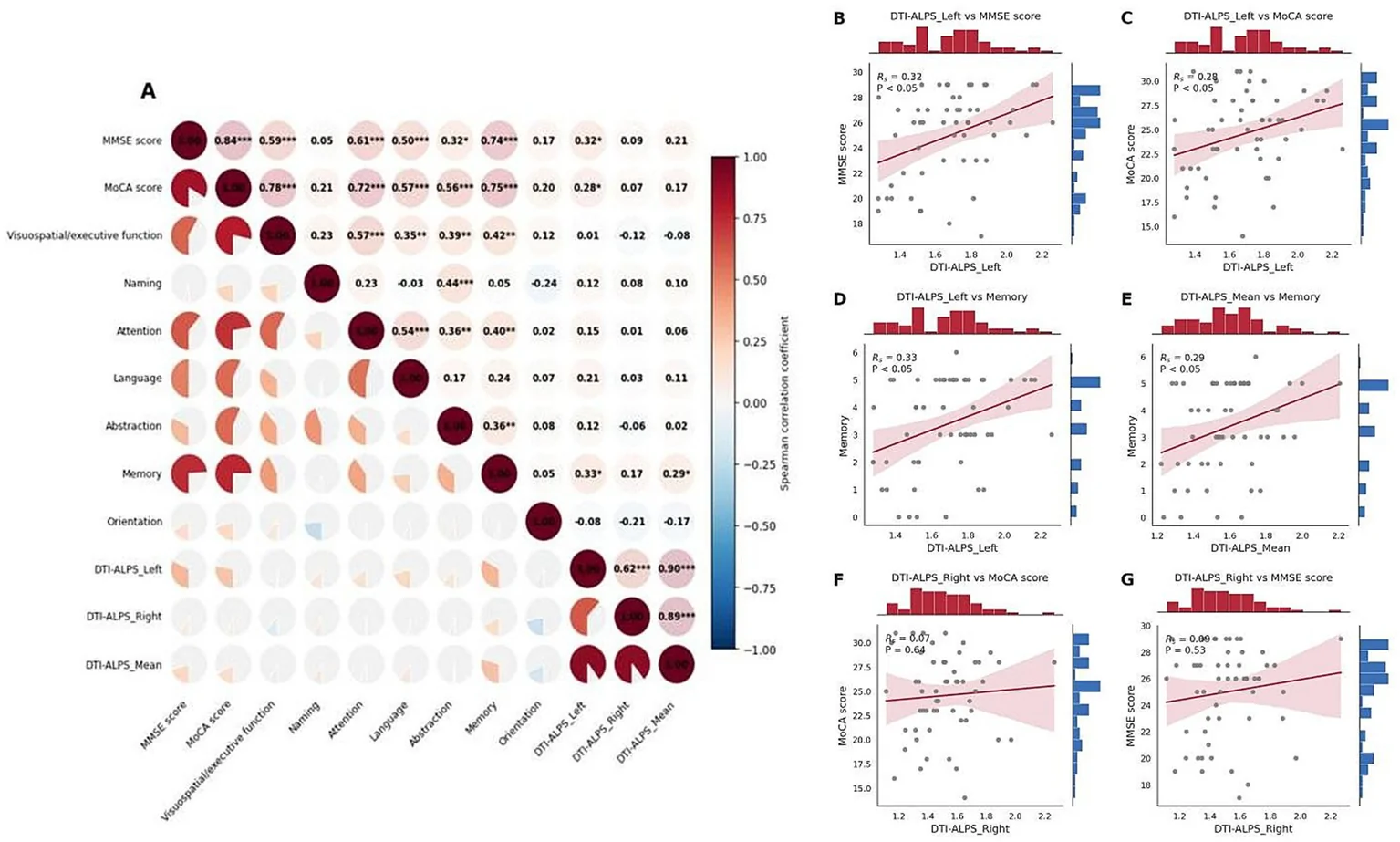
Correlation between DTI-ALPS index and cognitive performance in HFpEF patients. HFpEF, heart failure with preserved ejection fraction; DTI-ALPS, diffusion tensor image analysis along the perivascular space; MoCA: Montreal cognitive assessment; MMSE: mini-mental state examination. Panel **(A)** shows the correlation matrix of the DTI-ALPS indices and cognitive scores; panels **(B)–(E)** show the scatterplots for the statistically significant correlations; and panels **(F)** and **(G)** show the scatterplots of the right DTI-ALPS index against the total MoCA score and the MMSE score, respectively, neither of which was statistically significant.

The mean DTI-ALPS index was also positively associated with memory scores (*r* = 0.29, p < 0.05), whereas the right DTI-ALPS index was not significantly correlated with any of the cognitive scores (all p > 0.05), including the total MoCA score (*r* = 0.07, *p* = 0.64) and the MMSE score (*r* = 0.09, *p* = 0.53). The correlation matrix of DTI-ALPS indices and cognitive scores is shown in Figure 3A, association plots for the significant correlations are shown in Figures 3B–E, and the scatterplots of the right DTI-ALPS index against the total MoCA score and the MMSE score are shown in Figures 3F,G, respectively.

### 3.4 Multivariable associations between DTI-ALPS indices and cognitive function

Multivariable linear regression analyses were used to examine whether the left DTI-ALPS index was associated with cognitive performance after adjustment for age, sex, and educational level (Table 3). Higher left DTI-ALPS values were independently associated with higher MMSE scores (*β* = 0.031, SE = 0.009, *p* = 0.001, *R*^2^ = 0.222) and higher MoCA scores (*β* = 0.021, SE = 0.008, *p* = 0.007, *R*^2^ = 0.167).

**Table 3:** Associations between cognitive measures and DTI-ALPS_Left after adjustment.

Independent variable	*β*	SE	*p*value	*R* ^2^	*N*
MMSE score	0.031	0.009	0.001	0.222	55
MoCA score	0.021	0.008	0.007	0.167	55
Delayed recall (MoCA)	0.050	0.019	0.009	0.160	55

All models were adjusted for sex, age, and education level. MMSE, mini-mental state examination; MoCA, Montreal cognitive assessment; DTI-ALPS, diffusion tensor image analysis along the perivascular space.

The left DTI-ALPS index was also independently associated with memory performance (*β* = 0.050, SE = 0.019, *p* = 0.009, *R*^2^ = 0.160). These findings indicate that lower left DTI-ALPS values are associated with poorer cognitive performance in this cohort.

## 4 Discussion

In this study, patients with HFpEF demonstrated poorer cognitive performance than healthy controls. Deficits were observed in global cognitive measures and in several MoCA subdomains, including visuospatial/executive function, attention, language, abstraction, and memory. These findings suggest that cognitive impairment in HFpEF may involve multiple cognitive domains and may occur even in patients without clinically overt neurological disease, supporting the concept that HFpEF can be accompanied by subclinical brain involvement (2).

The pattern of cognitive impairment was not uniform across domains. Naming and orientation were relatively preserved, whereas executive, attentional, language, abstract reasoning, and memory-related functions were more affected. This pattern may be compatible with preferential vulnerability of frontal-subcortical and distributed network functions to chronic cerebral hypoperfusion, microvascular dysfunction, and metabolic stress. By contrast, preserved naming and orientation may indicate that certain temporal-limbic and semantic functions were relatively less affected in the current cohort. This selective profile should be interpreted cautiously because only screening-scale subdomains were available, but it provides a useful basis for future studies using comprehensive neuropsychological batteries.

Several cardio-cerebrovascular pathways may contribute to cognitive impairment in HFpEF. Chronic reductions in cardiac reserve, endothelial dysfunction, arterial stiffness, impaired cerebrovascular autoregulation, systemic inflammation, oxidative stress, and metabolic comorbidities may collectively increase cerebral vulnerability (3, 10, 11, 17). These pathways may promote microvascular injury, blood–brain barrier dysfunction, neuroinflammation, and disruption of neural network function. However, the present study was not designed to test these mechanisms directly, and these explanations should be considered hypothesis-generating rather than causal.

A notable finding was the left-predominant reduction in DTI-ALPS in patients with HFpEF. The left DTI-ALPS index was significantly lower in patients than in controls, while right-sided and mean indices were not significantly different. Several explanations may be considered. First, hemispheric differences in vascular anatomy, arterial pulsatility, venous drainage, or CSF-perivascular coupling could create asymmetric vulnerability to systemic hemodynamic stress. Second, the left hemisphere may have relatively high metabolic and vascular demands related to language and complex cognitive processing in most right-handed individuals (14), which could increase susceptibility to subtle perfusion or perivascular clearance abnormalities. Third, the absence of significant right-sided or mean differences may partly reflect limited statistical power rather than true preservation of right-sided perivascular function. Therefore, the lateralized pattern should be regarded as an exploratory observation requiring confirmation in larger cohorts.

The proposed link between HFpEF, glymphatic-perivascular dysfunction, and cognitive decline remains indirect. Arterial pulsation is an important driver of perivascular CSF movement (9), and HFpEF-related reductions in cardiac reserve or arterial compliance could theoretically attenuate perivascular fluid movement. Reduced clearance efficiency may in turn contribute to the accumulation of neurotoxic solutes and to neuronal dysfunction (7, 18–20). Nevertheless, we did not directly measure cardiac output, stroke volume, arterial stiffness, pulse-wave amplitude, cerebral perfusion, or CSF flow velocity. Thus, the present data demonstrate associations between HFpEF, DTI-ALPS-derived perivascular diffusivity, and cognition, but they do not establish a mechanistic pathway. Future multimodal studies combining echocardiography, arterial stiffness assessment, perfusion imaging, phase-contrast MRI, and DTI-ALPS are needed to test this hypothesis.

The association between lower left DTI-ALPS and poorer cognitive performance remained significant after adjustment for age, sex, and educational level. This suggests that altered perivascular diffusivity may represent a brain imaging correlate of cognitive impairment in HFpEF. However, the regression findings should not be interpreted as evidence that DTI-ALPS predicts cognitive decline, because the study was cross-sectional and did not include longitudinal cognitive outcomes. Larger prospective studies are needed to determine whether DTI-ALPS provides incremental prognostic information beyond conventional clinical and imaging measures.

The association with memory is also noteworthy. In the present study, memory was defined as the delayed recall subscore of the MoCA. Lower memory scores were observed in patients with HFpEF and were associated with DTI-ALPS measures. Previous studies have linked impaired glymphatic-perivascular function with cognitive decline and memory-related impairment in other populations (8, 21). Our results extend this concept to HFpEF, but the use of a single MoCA sub item limits the precision of memory assessment. Future work should include detailed episodic, working, and verbal memory tests.

These findings may have clinical implications. Cognitive impairment should be considered a relevant complication in HFpEF, and routine cognitive screening may help identify patients at risk for functional decline (4, 15). DTI-ALPS may provide a noninvasive imaging marker of perivascular diffusivity that complements conventional structural MRI. At the same time, clinical application will require validation in larger and longitudinal cohorts, standardization of image acquisition and ROI placement, and evaluation of whether DTI-ALPS adds value beyond established cardiovascular, neuroimaging, and cognitive measures.

Because this was a cross-sectional associative study, the temporal relationship among HFpEF, altered perivascular diffusivity, and cognitive impairment cannot be determined. It remains unclear whether HFpEF contributes to glymphatic-perivascular impairment, whether both arise from shared mechanisms such as inflammation or endothelial dysfunction, or whether reduced DTI-ALPS reflects downstream changes related to pre-existing brain vulnerability. Longitudinal studies with repeated cardiac, neuroimaging, and cognitive assessments are required to clarify these relationships.

## 5 Limitations

This study has several limitations. First, the cross-sectional design precludes inference regarding causality or temporal sequence among HFpEF, DTI-ALPS alterations, and cognitive impairment. Second, the sample size was modest, with 35 patients with HFpEF and 20 healthy controls. This may have reduced statistical power, increased the risk of type II error, and partly explain why the right-sided and mean DTI-ALPS indices did not reach statistical significance. The limited sample size may also reduce the stability and generalizability of the findings. Third, DTI-ALPS is an indirect and static surrogate of glymphatic-perivascular activity rather than a direct measure of lymphatic fluid dynamics, interstitial solute transport, or CSF flow. The index may be influenced by local white-matter microstructure, perivascular extracellular matrix composition, capillary density, motion, and ROI placement, and therefore should be interpreted primarily as a measure of perivascular diffusivity rather than definitive glymphatic function. Fourth, several potential confounders were not assessed or adjusted for, including sleep quality and architecture, medication use, disease duration, atrial fibrillation burden, cardiac output, stroke volume, arterial stiffness, cerebral perfusion, and CSF flow parameters. These unmeasured factors may have influenced both DTI-ALPS values and cognitive performance. Finally, the memory analysis was based on the MoCA delayed recall subscore rather than a comprehensive memory battery. Future multicenter longitudinal studies with larger samples and multimodal heart-brain assessment are needed.

## 6 Conclusion

Patients with HFpEF showed cognitive impairment and a left-predominant reduction in DTI-ALPS-derived perivascular diffusivity. Lower left DTI-ALPS values were associated with worse global cognitive performance and memory after adjustment for demographic factors. These findings support an association between HFpEF and early brain changes involving perivascular diffusivity, but they do not establish causality. Longitudinal multimodal studies are needed to determine whether altered DTI-ALPS precedes or contributes to cognitive decline in HFpEF.

## Data Availability

The original contributions presented in the study are included in the article. Further inquiries can be directed to the corresponding author.
